# Relevance of Pathogenetic Mechanisms to Clinical Effectiveness of B-Cell-Depleting Monoclonal Antibodies in Multiple Sclerosis

**DOI:** 10.3390/jcm11154288

**Published:** 2022-07-23

**Authors:** Luca Massacesi, Alice Mariottini, Ferdinando Nicoletti

**Affiliations:** 1 Department of Neurosciences, Drug and Child Health, University of Florence, 50139 Florence, Italy; luca.massacesi@unifi.it; 2 Department of Physiology and Pharmacology, University Sapienza of Rome, 00185 Roma, Italy; ferdinandonicoletti@hotmail.com; 3 IRCCS Neuromed, 86077 Pozzilli, Italy

**Keywords:** multiple sclerosis, B cell-depleting therapy, monoclonal antibody, compartmentalised inflammation

## Abstract

Evidence of the effectiveness of B-cell-depleting monoclonal antibodies (mAbs) in multiple sclerosis (MS) prompted a partial revisitation of the pathogenetic paradigm of the disease, which was, so far, considered a T-cell-mediated autoimmune disorder. Mechanisms underlying the efficacy of B-cell-depleting mAbs in MS are still unknown. However, they likely involve the impairment of pleiotropic B-cell functions different from antibody secretion, such as their role as antigen-presenting cells during both the primary immune response in the periphery and the secondary response within the central nervous system (CNS). A potential impact of B-cell-depleting mAbs on inflammation compartmentalised within the CNS was also suggested, but little is known about the mechanism underlying this latter phenomenon as no definite evidence was provided so far on the ability of mAbs to cross the blood–brain barrier and reliable biomarkers of compartmentalised inflammation are lacking. The present paper briefly summarises the immunopathogenesis of MS with a focus on onset of autoimmunity and compartmentalisation of the immune response; mechanisms mediating B-cell depletion and underlying the effectiveness of B-cell-depleting mAbs are also discussed.

## 1. Introduction

Multiple sclerosis (MS) is an inflammatory demyelinating autoimmune disease of the central nervous system (CNS) that typically manifests with episodes of new/recurrent neurological symptoms (i.e., relapses) followed by either complete remission or residual disability (relapsing–remitting, RR, course) [1]. In 10–15% of cases, progressive disability accrual, irrespective of relapses, occurs from disease onset: these cases are defined as primary progressive (PP) [2]. After an average of 10 to 20 years, most of the patients with RR-onset evolve towards a secondary progressive (SP) course which is characterised by subtle and progressive accumulation of disability independent of new focal inflammation (i.e., relapses and/or inflammatory lesions at magnetic resonance imaging—MRI) [2].

Distinctive clinical and radiological features characterise RR- and SP-MS: (i) RR-MS is characterised by the development of new lesions promoted by adaptive immunity, which may affect different areas of the CNS and are usually associated with the onset of new neurological symptoms congruous with the anatomical area involved (i.e., relapses); (ii) SP-MS is characterised by the persistence of chronic inflammation within the CNS compartment which acts beyond a closed or repaired blood–brain barrier (BBB), i.e., compartmentalisation of the inflammatory response [3]. This latter phenomenon contributes to ongoing demyelination and axonal damage within pre-existent inflammatory lesions and is associated with progressive deterioration in the already impaired functional system(s), with rare occurrence (or absence) of new lesions/symptoms. However, RR- and SP-MS might plausibly be placed at opposite ends of a continuum in the spectrum of disease mechanisms, with the inflammatory and degenerative phenomena underlying MS progression showing complex mutual interactions, each contributing to a variable extent across different stages of the disease [4].

Disease-modifying treatments (DMTs) currently approved for MS mostly target the immune system acting in the peripheral compartment, thus preventing the occurrence of new inflammatory lesions, whereas their potential impact on compartmentalised inflammation is still debated. Furthermore, no DMTs were proven to be effective in halting degenerative phenomena, nor in promoting re-myelination.

In the present manuscript, the pathogenesis of MS is briefly summarised with relevance to mechanisms of the effectiveness of DMTs focusing on B-cell-depleting therapies. Potential implications for therapeutic intervention are also discussed.

## 2. Insights into MS Pathogenesis: Onset of Autoimmunity

MS was traditionally considered a T-cell-mediated autoimmune disorder, based on preclinical data from animal models of the disease (experimental autoimmune encephalomyelitis—EAE) and evidence for T-cell infiltration in inflammatory lesions and normal-appearing white matter of autoptic and biopsy CNS specimens from affected individuals, with an association between CD8+ T cells number and axonal damage [5,6,7,8,9,10,11,12]. Furthermore, the identification of expanded T-cell clones in the brain parenchyma, cerebrospinal fluid (CSF), and peripheral blood of MS patients detected using T-cell receptor (TCR) analyses reinforced the hypothesis that inflammatory infiltrates were constituted by pathogenetic expanded T-cell clones reactive to myelin antigens [13,14,15,16]. Circulating CD4+ T cells from MS patients were indeed demonstrated to recognise myelin basic protein (MBP), proteolipid protein (PLP), and myelin oligodendrocyte glycoprotein (MOG), even if the same phenomenon was also observed in healthy individuals; evidence regarding potential differences between these groups in frequency and avidity of cell interactions is conflicting [17,18].

A contribution of B cells to MS pathogenesis was also suggested by preclinical and clinical evidence, and their role was recently re-evaluated with the observation of a remarkable therapeutic effect of B-cell-depleting strategies [19]. Innate immunity cells, including CNS-resident microglia, contribute to MS pathogenesis, and neurodegenerative phenomena possibly, at least in part, independent of inflammation play a role in advanced disease [3].

Although MS aetiology is unknown, several environmental and genetic risk factors were identified [20,21,22]. Class II major histocompatibility complex (MHCII) represents the major genetic risk factor, accounting for 20–30% of individual genetic susceptibility [22,23]. MHCII genes encode membrane glycoproteins that are expressed by professional antigen-presenting cells (APC), such as dendritic cells, macrophages, and B cells [24]. The MHCII complex plays a key role in the development of both primary and secondary T CD4-mediated immune response as it presents in its context small peptide antigens processed by professional APC to MHC-restricted T cells [25]. In addition to MHC, several other genes were associated with the risk of developing MS on the basis of a complex genetic background, as confirmed by genome-wide association studies (GWAS) that uncovered more than 200 genetic susceptibility variants which could jointly account for ~48% of the estimated heritability for MS [26]. Enrichment for MS susceptibility loci was mostly related to genes involved in immune system function and regulation, and it was apparent in many different immune cell types and tissues, including microglia, highlighting, overall, the relevance of adaptive and innate immune cells in MS pathogenesis.

### 2.1. Primary Autoimmune Response in the Peripheral Compartment

The mechanisms which trigger MS are still debated and two plausibly complementary pathogenetic models were proposed to explain the initiation of the autoimmune response, suggesting that the primum movens might take place either in the periphery (“outside-in” model) or within the CNS (“inside-out” model) [27].

The “outside-in” (or CNS-extrinsic/peripheral) model resembles the pathogenetic mechanism underlying EAE in which the disease is induced by external immunisation obtained through the inoculation of myelin-specific antigens in combination with an adjuvant [28]. According to this model, activation and expansion of CNS antigen-specific CD4+ T cells occur in the periphery and may be induced by an encounter with exogeneous antigens sharing structural motifs with myelin antigens, hence capable of eliciting an autoimmune response based on molecular mimicry [29,30]. Several infective agents were suggested as potential exogenous triggers of the autoimmune reaction, with Epstein–Barr virus (EBV) the most plausible candidate [31,32].

On the other hand, the “inside-out” model suggests that the autoimmune reaction is triggered by an “internal event” occurring within the CNS and generating myelin debris. CNS-derived soluble antigens may then be drained via lymphatic pathways to peripheral lymphoid organs, where they might be presented to T and B cells eliciting the autoimmune reaction [33,34]. Different hypotheses suggest that the CNS-intrinsic event might be triggered by a CNS viral infection or by primary neurodegeneration [11].

### 2.2. Secondary Autoimmune Response in the Central Nervous System

Once their priming has occurred, autoreactive T cells might be further expanded in peripheral lymphoid tissues after the encounter with their cognate antigen [22,35].

**Figure 1:**
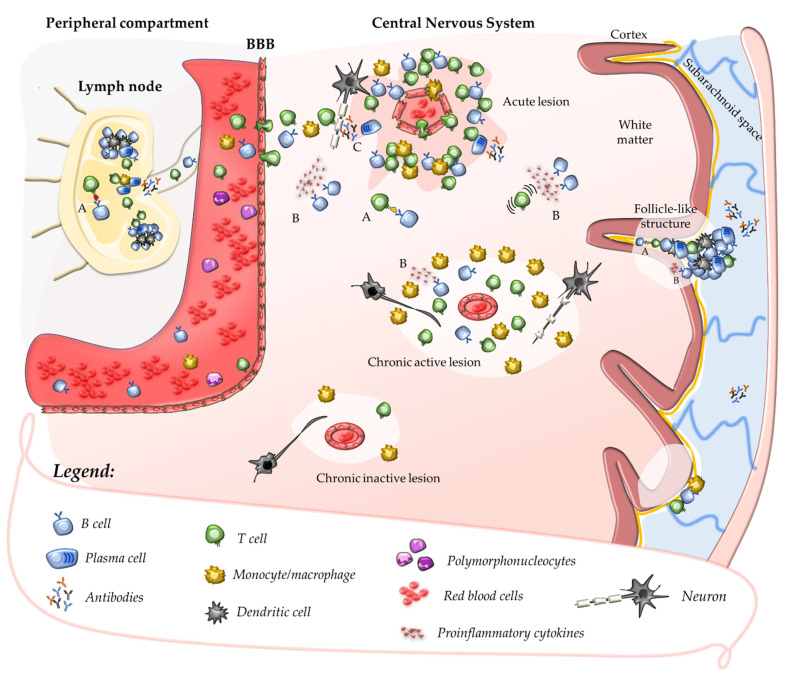
B cells play a pleiotropic role in MS pathogenesis. B lymphocytes act as antigen-presenting cells (A) in the periphery during the primary autoimmune response and may present CNS self-antigens to autoreactive T cells in lymph nodes. Once the autoimmune response is established, activated T and B cells and macrophages invade the CNS crossing the blood–brain-barrier (BBB) and promote the formation of acute inflammatory lesions that usually develop around small veins. MS acute lesions are characterised by a breakdown of the BBB of their central vein and dense perivenular inflammatory infiltrate. The secretion of pro-inflammatory cytokines by activated B cells (B) promotes the recruitment of inflammatory cells and their further activation. Antibody secretion (C) might contribute to demyelination and axonal damage, which are mostly T-cell mediated. Over the disease course, acute lesions may evolve towards chronic active lesions that are characterised by moderate–low grade inflammatory infiltrate, absence of macroscopic leakage of the BBB (compartmentalised inflammation) and a rim of macrophages at the lesion edges. Progressive demyelination and axonal loss take place within chronic active lesions, that tend to expand towards the surrounding normal-appearing white matter. In advanced MS, exhaustion of the inflammation and glial scarring eventually determine the transition from chronic active to chronic inactive lesions. During the course of the disease, inflammatory infiltrates containing B cells invade perivascular spaces of the leptomeninges and organise in follicle-like structures resembling tertiary lymphoid tissue. The release of soluble factors from such structures is thought to contribute to cortical pathology in the adjacent cortical grey matter.

In the absence of neuroinflammation, T cells patrol the CNS crossing the BBB at the level of CNS post-capillary venules and reaching perivascular or subarachnoid spaces [36]. In such areas, putative cross-reactive T-cell clones may be further activated by CNS antigens (sharing structural motifs with their cognate antigen) which, after being drained from the parenchyma, are processed and presented by tissue-resident APCs. This mechanism promotes a secondary immune response within the CNS characterised by cell proliferation, recruitment of pro-inflammatory cells, and formation of perivascular cuffs around post-capillary and pial venules [37]. Upon activation, adaptive immune cells cross the glia limitans and infiltrate the CNS parenchyma where they produce pro-inflammatory cytokines and chemokines that determine a breakdown of the BBB. This causes further recruitment of adaptive and innate immune cells, which ultimately promote the formation of demyelinating lesions and tissue injury (Figure 1) [37,38]. Further damage to CNS tissue might derive from uncovering and release in the extracellular space of several autoantigens which, in turn, might enhance the autoimmune response in a mechanism defined as epitope spreading [39].

The formation of new inflammatory lesions is frequent in RR-MS, in which subsequent “inflammatory waves” of autoimmune cells invade the CNS, and it is usually associated with the onset of new clinical symptoms (relapses) reflecting the impairment of the area involved. In early MS, relapses usually resolve without sequelae, thanks to functional compensation by the huge number of residual nerve fibres. The number and frequency of relapses occurring shortly after disease onset predict the accumulation of long-term disability and the achievement of pre-defined disability milestones, suggesting that in the RR phase acute inflammation is the main driver of disease activity and tissue damage [40]. Accordingly, disability worsening in RR-MS is mainly due to incomplete recovery from relapses, and no intercurrent disability progression is observed [2].

Acute inflammatory lesions are characterised by predominant demyelination with axonal injury but moderate axonal loss and gliosis and can be visualised by MRI as new hyperintense T2 lesions in the white matter of the CNS; acute lesions show gadolinium enhancement in the first weeks of their development, mirroring the breakdown of the BBB [41]. After the resolution of acute oedema, hyperintense T2 lesions shrink and evolve into iso- or hypointense T1 lesions, depending on the grade of residual tissue damage, the latter case being defined as “black holes” [42].

### 2.3. Contribution of B Cells and Humoral Response to Acute CNS Injury

The role of B cells and humoral immunity in the pathogenesis of MS was considered less prominent compared with that of T cells, possibly due to the lack of consistency in detecting antibodies specific to CNS self-antigens in brain lesions or CSF [43]. However, their contribution was recently reinforced by the observation of the remarkable effectiveness of monoclonal antibodies (mAbs) targeting this cell population [44].

Evidence for oligoclonal antibody production in the CSF dates to the 1940s [45], and an oligoclonal pattern of intrathecal production of immunoglobulins (OCBs) is detected in the CSF of the vast majority of MS patients; OCBs are plausibly produced by a restricted number of plasma cell clones recruited in the CNS during the secondary autoimmune response [46].

Experimental data derived from one of the animal models of MS more similar to humans—EAE induced by immunisation with myelin-antigens in marmosets—show that a secondary antigen-specific humoral immune response is elicited at a perivenular level by cellular immunity [47]. In the animal model, breakdown of the BBB in the surrounding capillaries is promoted by diffusive molecules secreted by immune cells aggregated as perivascular cuffs: in this setting, circulating antibodies specific to myelin antigens contribute to myelin damage [48]. However, differently from this model, circulating antibodies against CNS antigens are rarely detected in humans [49]; nevertheless, anatomopathological studies showing the deposition of immunoglobulins (mainly IgG) and complement C9neo antigen at sites of active myelin destruction in pattern II lesions suggest a possible contribution of the humoral response to tissue injury [50].

Pleiotropic roles of B cells independent of antibody secretion may be relevant to MS pathogenesis, such as antigen-presenting function or secretion of pro-inflammatory cytokines (Figure 1) [51]. Clonally expanded B-cell clones were detected in the meninges, parenchyma, and CSF of MS patients; they may indefinitely persist within perivascular and leptomeningeal inflammatory infiltrates where they organise in follicle-like tertiary lymphoid structures of aggregated plasma cells, B cells, T cells, and follicular dendritic cells [52,53,54]. Recent data suggest that B-cell accumulation within inflammatory infiltrates may be critical to the chronicisation of the intrathecal autoimmune response, acting as professional APCs for definite CNS autoantigens [55].

## 3. Compartmentalisation of the Inflammatory Response within the CNS

After the acute phase, the inflammatory infiltrates formed within the CNS parenchyma and perivascular spaces may encounter different destinies: (i) resolution without inducing relevant axonal damage or reactive gliosis, thus allowing the repair and re-myelination of the affected area, a phenomenon that is more efficient in young patients with short disease duration [56]; or (ii) organisation as chronic aggregates that may resemble tertiary lymphoid organs, constituted by CD4+ and CD8+ T cells, B cells, plasma cells, and dendritic cells [53,54]. These chronic aggregates persist within the CNS and become “independent” of the periphery, acting beyond a partially “closed” or repaired BBB, and are referred to as compartmentalised inflammation [57]. Anatomopathological hallmarks of compartmentalised inflammation encompass chronic perivascular cuffs in the cerebral white matter and pial vessels (follicle-like structures), and smouldering lesions (Figure 1).

### 3.1. Chronic Perivascular Cuffs and Smouldering Lesions

Inflammatory infiltrates that persist within perivenular spaces in demyelinated white-matter lesions maintain a chronic low-grade inflammatory state without macroscopic leakage of the BBB [50]. Chronic inflammation promotes subtle ongoing tissue demyelination and injury acting as “fire under ash”, possibly sustained by a continuous replenishment of CNS antigens from the interstitial fluid and CSF that are presented to T lymphocytes by resident APCs, possibly including B cells. This suggests a potential efficacy of B-cell-depleting strategies in such phenomenon.

At the anatomopathological level, chronic active inflammatory lesions are characterised by a hypo-cellular core with a paucity of inflammatory infiltrates and a rim of iron-laden macrophages/activated microglia at the lesion edges; these progressively expand towards the surrounding normal-appearing white matter, hence defined as smouldering lesions or slowly expanding lesions (SELs) [58,59]. The proportion of SELs increases over time during the disease course and is higher in progressive vs. relapsing disease, being rare in the acute monophasic disease and RR phase; on the other hand, they represented around 20% of the lesions analysed in SP-MS and PP-MS cases or cases with a disease duration longer than 30 years (23%) [58]. Furthermore, a higher proportion of mixed active/inactive lesions and a higher lesion load at the time of death were reported in patients who had a more severe disease course [60].

The pathogenetic mechanisms underlying SELs, where inflammation persists for several years promoting ongoing axonal damage and tissue loss within the same functional area [57,61], might explain the typical clinical picture of progressive MS patients who show a steady progression of pre-existing neurological symptoms, rather than the occurrence of new focal neurological dysfunction, which is instead typical in RR-MS. The threshold for clinical manifestations may be different depending on several factors, such as the area involved and the individual functional reserve.

According to these observations, SELs might be an anatomopathological hallmark of progression independent of relapse activity (PIRA) or silent progression, i.e., progression which occurs in the absence of clinical and radiological signs of inflammatory activity, a phenomenon typical of progressive MS that may also be detected in the early disease course [62].

### 3.2. MRI Markers of Compartmentalised Inflammation

Visualisation of a paramagnetic rim at the edges of hyperintense T2 lesions using susceptibility-weighted imaging MRI (paramagnetic rim lesions, PRL) was suggested to identify the rim of iron-laden macrophages described at the edges of SELs by anatomopathological studies [42,63]. Coupled with the observation that PRL showed a trend towards expansion, while rim negative lesions showed a trend towards shrinkage, it was suggested that PRL could be a biomarker of SELs and compartmentalised inflammation [64]. Identification of PRL, also with 3T MRI, allowed exploration of their presence and frequency in the clinical setting, and investigation into whether PRL might be a marker of response to treatment [65].

Chronic active lesions are also characterised by failure of re-myelination and ongoing axonal damage. These phenomena can be estimated in vivo by assessing the increase in T1 hypo-intensity over longitudinal follow-up MRI, as intralesional reduced intensity in T1-weighted sequences (i.e., black holes) was correlated to the extent of demyelination and axonal loss in biopsy specimens [66]; T1 hypointense lesion load and accumulation of black holes were also associated with disability and disease progression [67]. A study evaluating longitudinal MRI scans from 1889 patients in the ORATORIO and OPERA I-II trials showed that PP-MS patients had higher numbers of expanding lesions compared with relapsing patients, and that expanding lesions had a significantly lower T1 intensity at baseline and a larger decrease in T1 intensity over time compared with areas of T2 lesions not classified as expanding [68]. These observations corroborated the hypothesis that expanding lesions could represent a radiological correlate of the chronic active lesion observed in the anatomopathological studies, as progressive tissue loss was ongoing in their context.

Consistently, a reduction in T1 intensity over time was observed in a higher percentage of PRL compared with lesions without rim (70% vs. 27%, respectively, *p* = 0.03) in another study, where anatomopathological observation from an autopsy case showed that lesions with PRL were characterised by the presence of iron-laden CD68 positive cells at lesion edges; the authors suggested that the formation of a phase rim might be due to a lack of shift of activated macrophages/microglia from a pro-inflammatory to an anti-inflammatory phenotype, thus inducing the persistence of ongoing demyelination and chronic inflammatory activity at the lesion edges. Various individual factors might contribute to this phenomenon, and age at lesion formation appeared to be one of the prominent factors in inducing rim persistence [42].

### 3.3. Follicle-like Structures in the Leptomeninges

Infiltrates of inflammatory cells, mainly T and B lymphocytes and plasma cells, were described in the leptomeninges of MS patients [69], being aggregated as ectopic tertiary lymphoid structures and morphologically resembling lymphoid follicle-like structures in a subgroup of post-mortem acute and progressive MS cases with more rapid and severe disease progression [53]. Approximately 40% of the examined SP-MS cases harboured at least one detectable follicle-like structure in their forebrain meninges, mainly containing aggregates of B cells interacting with a network of follicular dendritic cells, and with T cells and plasma cells [70]. In anatomopathological studies, the presence of follicle-like structures was correlated with the extent of demyelination in the adjacent subpial cortical grey matter with a “surface-in” gradient of neurodegeneration and microglial activation, and with a more severe and rapid disease progression [54,71,72].

Leptomeningeal enhancement (LME) detected by MRI using post-contrast fluid-attenuated inversion recovery (FLAIR) images was suggested as an imaging marker of follicle-like structures [73]. However, LME is a non-specific finding showing different patterns possibly linked to different anatomopathological entities. It can be observed in several inflammatory and non-inflammatory neurological conditions, including stroke where it was associated with early BBB disruption [74]. In MS, LME is described from the early stages of the RR phase, but the number of LME increases with disease duration and transition to SP-MS [75,76]. The presence of LME was independently associated with grey matter injury using 7T MRI [77], although such association was not consistently described in another study [78]. A recent meta-analysis confirmed that the presence of LME in MS was associated with worse physical disability and higher lesion burden, as well as lower cortical volumes, substantiating its role as a prognostic biomarker in MS [79].

The presence of post-contrast enhancement suggests a BBB dysfunction in the meningeal vessel associated with follicle-like structures, raising the hypothesis that inflammatory cells residing in such areas may be affected by DMTs administered systemically; however, no suggestion of the effectiveness of currently available DMTs on LME was provided thus far [79].

### 3.4. Innate Immunity and Degenerative Phenomena

In addition to adaptive immune cells, dysregulation of innate immunity might play a role in MS pathogenesis [80,81]; innate immunity cells (including microglia) switched to activated phenotypes contribute to tissue injury with direct cell-to cell-interaction and the release of soluble factors, such as reactive oxygen and nitrogen species produced by oxidative burst [82]. This latter process may induce the accumulation of mutations in mitochondrial DNA, thus resulting in mitochondrial dysfunction and energetic failure. These phenomena could exert an even more detrimental effect in partially demyelinated axons that have undergone a redistribution of ionic channels and promote intracellular accumulation of calcium and potentially pro-apoptotic changes [83]. Subsequent exhaustion of inflammation and evolution to chronic inactive lesions is associated with glial scarring, determining marked and irreversible axonal damage [3].

In advanced disease, neurodegenerative phenomena contribute to tissue injury and possibly represent the main driver of irreversible disability accrual in late MS [3]. It is still a matter of debate whether or not neurodegeneration is a process separate from inflammation [84,85], as the mutual relationship between these two processes is yet to be elucidated [3]. Recent observations suggest that brain atrophy and disability accrual start early in the disease course and that, in RR-MS, progression of disability might occur even in the absence of clinical and radiological signs of inflammatory activity, a phenomenon defined as PIRA or “silent progression” (see above).

## 4. Relevance of Pathogenetic Mechanisms to the Effectiveness of Disease-Modifying Treatments

Both adaptive and innate immunity play a role in MS pathogenesis, and their activation towards pro-inflammatory phenotypes seems crucial to the development and maintenance of the auto-reactive immune response. Once autoimmunity is established, two different inflammatory processes can be observed: acute and chronic inflammation. Acute inflammation, predominant in the RR phase, is associated with the formation of new demyelinating lesions with BBB disruption lasting for a few weeks and acute tissue injury initiated by perivenular inflammatory infiltrates. New lesions may clinically manifest as relapse and often undergo tissue repair and re-myelination, especially in young patients. In this phase, dominated by the activation of adaptive immunity and formation of new demyelinating lesions, DMTs acting in the peripheral compartment (irrespective of their bioavailability within the CNS) can effectively prevent new inflammatory disease activity and disability accrual. They do this through the inhibition of autoreactive T cells activation and/or proliferation (either by a cytostatic or cytolytic mechanism, or a more selective inhibition of their function), or their invasion of the CNS (e.g., inhibition of BBB diapedesis, or egress from secondary lymphoid organs) [86].

In long-standing MS, the frequency of new CNS lesions gradually decreases, and chronic lesions become predominant over acute lesions in SP-MS [58]. Chronic inflammation consists of low-density inflammatory infiltrates compartmentalised beyond a repaired BBB that slowly promote ongoing tissue injury within SELs, which show a trend towards enlargement and confluency. From the clinical perspective, relapses occur less often than in the RR phase, and subtle progression of disability occurs in the absence of clinical and radiological signs of inflammatory activity [62]. Anti-inflammatory treatments may efficiently target chronic inflammation within SELs; however, as inflammation is compartmentalised beyond a macroscopically repaired BBB, the ability of a DMT to cross the BBB and act within the CNS compartment would be a prerequisite for reaching such inflammation. Furthermore, the validation of biomarkers that could assess in vivo the evolution of SELs (such as PRL, or enlargement of T1 hypointense lesions) is required to properly assess the effectiveness of such treatments [68]; this is because PRL detected at MRI may not reliably distinguish between chronic active and inactive lesions.

In addition to inflammation, degenerative phenomena contribute to tissue injury, mostly in progressive phases, but anti-inflammatory treatments are not assumed to halt those processes that are independent of inflammation. In this regard, despite the fact that some DMTs used in MS were shown to be able to cross the BBB and exert neuroprotective functions in preclinical studies, the treatment of neurodegeneration in advanced MS is still an unmet clinical need. Similar to other degenerative diseases of the CNS, it may indeed not be possible to halt neurodegenerative phenomena once they have already started, nor to effectively prompt regeneration of functional neurons. Consequently, preventing the formation of chronic lesions and tissue injury is of paramount importance in the management of patients with MS, and this target should be pursued from the early stage of the disease when irreversible disability has not yet developed, even at the cost of accepting a less favourable safety profile of high efficacy DMTs.

## 5. B Cells and B-Cell-Depleting Antibodies

### 5.1. B-Cell Maturation and Surface Markers

B lymphocyte maturation occurs within the bone marrow, where haematopoietic stem cells differentiate into pro-B cells expressing the CD19 surface marker, but not CD20 or immunoglobulins (Ig) G type 1. This Ig subclass mainly recognises protein antigens and induces complement activation through the classic pathway, and opsonisation. Pro-B cells then mature into pre-B cells which express the marker CD20 (CD19^+^CD20^+^Ig^−^), and the subsequent transition to mature B cells is associated with the expression of surface Ig (CD19^+^CD20^+^Ig^+^ cells). Once migrated to peripheral lymphoid organs, immature B cells generate naïve B cells (CD19^+^CD20^+^Ig^−^CD38^+/−^) that can further differentiate into activated B naïve cells (CD19^+^CD20^+^Ig^−^CD38^+^), germinal centre (GC) B cells (CD19^+^CD20^+^Ig^−^CD38^++^), post-GC B cells (CD19^+^CD20^+^Ig^−^CD38^+^), and memory B cells (CD19^+^CD20^+^Ig^+/−^CD27^+^CD38^−^). Memory B cells generate plasmablasts which do not express CD20 (CD19^+^CD20^−^Ig^+/−^CD27^++^CD38^++^), and finally plasma cells (CD19^+/−^CD20^−^Ig CD27^++^CD38^+++^CD138^+^) [87].

Surface markers expressed by B lymphocytes are all potential therapeutic targets for the treatment of autoimmune neurological diseases, such as CD20, CD19, B-cell activating factor (BAFF) receptor (interacting with the Blyss protein), transmembrane activator and CAML interactor (TACI, interacting with the protein—a proliferation-inducing ligand—APRIL), and CD22. Three mAbs directed against the CD20 antigen (ocrelizumab, ofatumumab, and rituximab, the latter as off-label treatment) are used in the treatment of MS [88]. An anti-CD19 mAb (inebilizumab) is adopted for the treatment of neuromyelitis optica spectrum disorder (NMOSD) [89].

In MS, the CD20 antigen is a more appropriate therapeutic target than the CD19 because treatment is mainly aimed at preventing antigen presentation to T cells, and/or the release of pro-inflammatory cytokines from activated B cells, rather than halting the production of antigen-specific antibodies from late plasmablasts and plasma cells. Furthermore, the absence of the CD20 marker in stem cells and pro-B cells allows the repletion of B cells when the treatment with CD20-depleting mAbs is discontinued, although the efficacy of CD20-depleting mAbs in MS persists after the last drug administration. This may be due to long-term modification of immune cell subsets, such as the increase in naïve and immature B cells (CD5CD38^high^), the reduction in autoreactive T-cell phenotypes (such as Th1 and Th17), and an increase in T regs (CD25^+^FOXP3^+^) during immune-cell repopulation [51].

On the other hand, the rationale for the use of anti-CD19 mAbs in NMOSD is based on evidence of a direct pathogenetic role of anti-AQP4 antibodies produced by late plasmablasts (expressing CD19, but not CD20). Other potential targets for the treatment of NMOSD were suggested, such as the interleukin 6 (IL-6) receptor and the C5 component of the complement cascade. MAbs directed against IL-6 receptor and C5 showed high efficacy and an acceptable safety profile in phase 3 clinical trials [90,91].

### 5.2. Mechanisms of Lymphocyte Depletion Induced by Anti-CD20 Monoclonal Antibodies

Three anti-CD20 mAbs are currently used for the treatment of MS (rituximab, ocrelizumab, and ofatumumab), whereas another anti-CD20 mAb (obinutuzumab) is available with other therapeutic indications [92].

Biological peculiarities of mAbs are indicated by their denomination: the last three letters (mab) indicate their monoclonal nature (mab = monoclonal antibody). The preceding two letters indicate the nature of the Ab: “xi” (rituximab) for chimeric, i.e., merge of a human constant portion and a murine variable portion; “zu” (ocrelizumab) for humanised Ab, i.e., only the hypervariable domain recognizing the antigen is produced by murine cells; “u” (ofatumumab), i.e., fully human. All three mAbs show a high affinity and specificity towards the CD20 antigen, although they differ widely with respect to their pharmacodynamic profile [88].

The CD20 protein is composed of four trans-membrane domains (TM) that are connected by two extracellular loops (between the TM1 and TM3, and between the TM3 and TM4, respectively) and by one intracellular loop (between TM2 and TM3). The N-terminal and C-terminal portions of the protein are both intracellular.

Rituximab and ocrelizumab bind two partially overlapping epitopes localised in the intermediate-distal portion of the second extracellular loop [93].

Amino acids 170-172 are essential for the binding of both rituximab and ocrelizumab, whereas amino acids 162-166 constitute a second binding site specific to ocrelizumab. A completely different binding site is recognised by ofatumumab, and is localised between the first extracellular loop and the proximal portion of the second extracellular loop. Different binding sites offer alternative therapeutic options to patients showing genetic variants of the CD20 marker; although rare, genetic mutations of the epitopes of CD20 were described in patients affected by Hodgkin’s lymphoma showing resistance to rituximab [94,95].

The CD20 surface marker is expressed from intermediate stages of B-cell maturation (from pre-B cells to plasmablasts). The biological function of the protein CD20 is not fully elucidated but experimental data suggest that it contributes to the increase in intracellular levels of calcium following B-cell receptor activation; however the role of this mechanism in the process of B-cell activation is still debated [96]. The therapeutic effect of anti CD20-depleting mAbs in MS is plausibly mediated by B-cell depletion rather than by the functional inhibition of the CD20 molecule.

B-cell depletion may occur via four different mechanisms: (i) complement-mediated cytotoxicity; (ii) antibody-mediated cell cytotoxicity; (iii) antibody-mediated cell phagocytosis; (iv) direct cytotoxicity mediated by irreversible cytoskeletal damage, inappropriately denominated “direct apoptosis” [97].

Ocrelizumab is an IgG4 mAb highly effective in promoting antibody-mediated cytotoxicity and phagocytosis, but less active than rituximab in promoting complement-mediated cell lysis. This difference accounts for the lower rate of infusion-related reactions observed with ocrelizumab compared with rituximab, as such reactions are mediated by complement activation [88]. The different administration route of ofatumumab (i.e., subcutaneous) does not allow a direct comparison of its administration-related adverse reactions [98].

Both antibody-mediated cell cytotoxicity and phagocytosis depend on an interaction between the constant portion (Fc) of the mAb and the FcγRIIIa receptor expressed by NK cells and macrophages. FcγRIIIa is a polymorphic receptor that may present either a residual phenylalanine (Phe) or valine (Val) in position 158. The 158Phe variant has low-affinity and is predominant in the population with respect to the high-affinity variant, 158Val. The effectiveness of rituximab in haematological diseases is influenced by the genetical variant of FcγRIIIa and the presence of the 158Phe variant may confer resistance to treatment. Ocrelizumab binds to FcγRIIIa with high efficacy and its action is not influenced by Phe158Val polymorphism.

Ocrelizumab and rituximab, therefore, show remarkable differences in their pharmacodynamic profiles deriving from the differences in their primary structures; these make ocrelizumab less prone to induce infusion-related reactions, and plausibly more effective in patients who show a reduced response to rituximab caused by the presence of the FcγRIIIa 158Phe polymorphism. Both ocrelizumab and ofatumumab can induce the “direct” apoptosis of B cells [93].

Another relevant biological difference between ocrelizumab and rituximab is due to the nature of the two mAbs, i.e., humanised and chimeric, respectively. The production of anti-rituximab antibodies is observed in 36% of treated patients affected by RR-MS and 26% of those affected by progressive MS [99]. On the other hand, the proportion of those developing anti-mAb antibodies is low in ocrelizumab-treated patients: 0.4 and 1.9%, respectively [100]. Anti-Ab antibody production is negligible in patients treated with ofatumumab, which is constituted by human sequences only.

### 5.3. Immunological Effects of B-Cell Depletion

Several mechanisms might underlie the therapeutic effectiveness of anti-CD20 mAbs; the relevance for mechanisms different from the impairment of antibody-secretion due to the elimination of precursors of plasma cells was suggested by observations derived from the first clinical study on rituximab in MS [101]. There was little expectation of success in this trial because plasma cells do not express the CD20 marker: they are long-lived with sustained production of antibodies, as demonstrated by prior observations that IgG levels were unchanged in most of the rituximab-treated patients, with IgM only being reduced [102]. This observation could, therefore, suggest that several years of treatment would be required to observe a clinically meaningful effect. In contrast, rituximab exerted a rapid and remarkable effect on the primary endpoint (inflammatory lesions detected by MRI) and on clinical relapses, suggesting that its clinical effectiveness could be mediated by mechanisms independent of plasma cells or antibodies depletion, although a potential contribution of IgG4 reduction cannot be excluded [103].

The effectiveness of B-cell depletion in MS is considered to be mediated, at least in part, by inhibition of their antigen-presenting function (which may be pivotal in the presentation of CNS self-antigens), and by the removal of pro-inflammatory B-cell phenotypes with a subsequent reshaping of the B-cell population towards an anti-inflammatory milieu [44,55,104]. B cells are efficient APC to T lymphocytes, and marginal zone B cells residing in secondary lymphoid organs can capture and deliver systemic antigens to follicular dendritic cells through their marginal zone-follicle shuttling [105]. The impairment of such mechanisms induced by B-cell-depleting mAbs may contribute to their therapeutic effect (Table 1).

Furthermore, a subpopulation of T cells expresses the surface marker CD20 at low levels (defined as CD3^+^CD20^dim^); this population represents about 7% of the total T-cell population and includes both CD4+ and CD8+ T lymphocytes [106], but it is still unknown whether its depletion might contribute to the clinical effectiveness of anti-CD20 mAbs in MS and other autoimmune diseases.

### 5.4. Impact of CD20-Depleting Monoclonal Antibodies on the Peripheral Autoimmune Response and Compartmentalised Inflammation

The abovementioned mechanisms are relevant mostly for the suppression of the peripheral autoimmune response and the secondary autoimmune response within the CNS in the acute phase of newly forming lesions, where the breakdown of the BBB might allow migration of mAbs to inflammatory infiltrates (Table 1). In addition, the maturation of antigen-experienced B cells in draining cervical lymph nodes before their transmigration to the CNS [107] might support the rationale of treatments targeting B cells in the peripheral compartment [101,108].

On the other hand, the absence of overt damage of the BBB in chronic lesions likely prevents the transfer of high molecular weight molecules, such as rituximab, ocrelizumab, and ofatumumab [109]. Data from haematological patients show poor penetration of rituximab across the BBB, CSF levels being 0.1% of plasma levels [110,111]. According to these hypotheses, if suppression of the APC function of B cells occurred exclusively in the peripheral compartment (i.e., during the first stage of autoimmune reaction), no effect can be expected on compartmentalised inflammation which dominates the progressive and advanced stages of MS. However, ocrelizumab showed efficacy in PP-MS, this event being one of the most relevant breakthroughs in the treatment of MS in recent years [112]. Furthermore, during ocrelizumab therapy, a reduction in the enlargement of T1 hypointense lesions possibly corresponding to SELs was observed both in PP- and RR-MS [68,113]. However, the mechanism underlying the effectiveness of B-cell depletion in PP-MS is yet to be elucidated; it is also unclear whether these mAbs can be effective in SP-MS. It may be tempting to speculate that such molecules can achieve their target within the leptomeningeal follicle-like structures where the BEE is dysfunctional, or that they might migrate to CNS parenchyma through the BBB in areas involved by smouldering inflammation, even in the absence of gadolinium leakage; indeed, BBB dysfunction with increased permeability was previously suggested in the normal-appearing white matter of MS patients compared with healthy controls [114]. The delivery of mAbs to sites of active inflammation (such as SELs) through a permissive BBB might be relevant for their effectiveness, as intrathecal administration of rituximab showed little effect on CSF markers of inflammation and tissue damage, without remarkable modification in clinical outcome and biomarkers of inflammation, including LME [115,116].

The ability of mAbs to cross the BBB may depend on several factors, such as their intrinsic structural characteristics or their plasma concentrations at peak and steady state, this latter influenced by the dose and route of administration, the volume of distribution, and mechanisms of clearance of the mAb. In this respect, comparative studies between rituximab, ocrelizumab, and ofatumumab could show differential activity in the intrathecal compartment and SELs, potentially uncovering different profiles of efficacy in PP- or advanced MS.

To our knowledge, little data are available on the potential impact of B-cell-depleting mAbs on CNS resident cells other than lymphocytes. Data from experimental models suggested that B-cell-depleting mAbs could reduce astrocyte and microglial activation within MS-like lesions and extralesional CNS tissue, these effects likely being mediated by the reduction in B and T cells infiltrating the CNS [117,118].

**Table 1:** Binding site of B-cell-depleting antibodies used for the treatment of MS and supposed mechanisms mediating their impact on acute and chronic inflammation.

	Rituximab	Ocrelizumab	Ofatumumab
Binding site of the CD20 protein	intermediate-distal portion of the extracellular loop between TM3 and TM4 (amino acids 170–172)	intermediate-distal portion of the extracellular loop between TM3 and TM4 (amino acids 170–172 + 162–166)	between the first extracellular loop and the proximal portion of the second extracellular loop
Acute focal inflammation	Depletion of potentially pathogenetic B and CD20+ T cells. Impairment of antigen-presentation in peripheral lymphoid organs (primary autoimmune response) and within the CNS (secondary autoimmune response). Reduced production of B-cell-derived pro-inflammatory cytokines.		
Chronic inflammation compartmentalised within the CNS	Reduced replenishment of encephalitogenic cells from peripheral blood. Impairment of antigen-presentation within the CNS (maintenance of the secondary autoimmune response and epitope-spreading). Reduced production of B-cell-derived pro-inflammatory cytokines. Reduced activation of microglia and astrocytes. ^a^ Depletion of B cells from follicle-like structures in the meninges. ^a^		

TM: transmembrane domain. ^a^ pre-clinical evidence [117,118,119].

## 6. Conclusions

The mechanisms underlying the suppression of inflammatory activity induced by B-cell depletion are only partially known and possibly involve pleiotropic roles of B cells, being the impairment of the antigen-presenting function the most plausible candidate. However, it is not clear yet if B-cell-depleting mAbs can act only in the peripheral compartment or if they also affect compartmentalised inflammation, as may be suggested by evidence of efficacy in progressive disease. Further knowledge of the impact of anti-CD20 mAbs on compartmentalised inflammation and of mechanisms underlying their potential effectiveness is needed to promote a tailored therapeutic approach, possibly offering additional treatment opportunities in progressive MS, an area where a remarkable unmet clinical need persists.
